# Supplementary material for: Harm reduction strategies in acute care for people who use alcohol and/or drugs: A scoping review
Source: PLoS One. 2023 Dec 15;18(12):e0294804. doi: 10.1371/journal.pone.0294804 (PMC10723714; doi:10.1371/journal.pone.0294804)
Supplement: S3 Table — (DOCX) [file pone.0294804.s003.docx]

**S3 Table. The Guidance for Reporting Involvement of Patients and the Public revised short form (GRIPP2-SF) checklist.**

| **Section and topic** | **Item** | **Reported on page No** |
| --- | --- | --- |
| 1: Aim | Report the aim of PPI in the study | Page 5-6 |
| 2: Methods | Provide a clear description of the methods used for PPI in the study | Page 5-6 |
| 3: Study results | Outcomes—Report the results of PPI in the study, including both positive and negative outcomes | Page 6 |
| 4: Discussion and conclusions | Outcomes—Comment on the extent to which PPI influenced the study overall. Describe positive and negative effects | Page 22-24 |
| 5: Reflections/critical perspective | Comment critically on the study, reflecting on the things that went well and those that did not, so others can learn from this experience | Page 24 |
