# Supplementary material for: Harm reduction strategies in acute care for people who use alcohol and/or drugs: A scoping review
Source: PLoS One. 2023 Dec 15;18(12):e0294804. doi: 10.1371/journal.pone.0294804 (PMC10723714; doi:10.1371/journal.pone.0294804)
Supplement: Attachment — Submitted filename: Response to Reviewers.docx [file pone.0294804.s004.docx]

| **Reviewer #1 Comments** | **Authors’ Response** |
| --- | --- |
| Given the ongoing morbidity and mortality caused by substance use, there has been a push to look at novel ways to engage People Who Use Alcohol and/or Drugs (PWUAD). Many of such novel approaches are based in the ER as PWUAD frequently show up there to seek care as they do not have a steady source of income or Insurance (in the USA). Unfortunately, this has also led to some ER physicians and employees developing a stigma against PWUAD. This has been observed by this reviewer on many occasions while talking to MDs not trained in psychiatry. The authors can consider discussing this as a potential motivating factor for healthcare providers reporting a lack of training in addictions as a more palatable reason to refuse providing care. Tackling this stigma is key to increase willingness to implement such strategies mentioned in line 305. | Thank you for this comment. We agree that stigma toward PWUAD by providers is a complex issue which should be addressed. We have highlighted the potential for strategies, such as training, in EDs on line 359-362 |
| The authors can consider adding a comment about the recent decision by the DEA to drop the X waiver requirement. | A comment about the X-waiver decision has been added to the introduction (page 3, line 74-77) |
| The authors can also consider providing housing resources as harm reduction strategies being used in other settings mentioned in line 313. | This sentence was amended to include a reference related to providing housing as a harm reduction strategy (page 24, line 366) |
| This article is a great teaching tool to be discussed in schools and residency training programs to ensure healthcare providers are provided education at a stage before they start working as attendings and develop stigma. The authors can consider tabulating the points mentioned from line 354 to 370 as these are great teaching points for a residency program. | To improve readability the Partner Reflections on Gaps in the Literature section has been placed in a table and has been moved before the discussion. (page 23, line 325-326) |
| Line 22- please consider rephrasing, the current sentence reads awkwardly. | This sentenced has been amended for clarity (page 2, line 21-23) |
| Line 52- there should be a / in between "and" and "or" like was mentioned in line 21. | A “/” has been added between the “and” and “or” (page 3, line 57) |
| Line 55- the comma can be removed. | This comma has been removed (page 3, line 59) |
| Line 74 and 75- just because PWUAD presents to the hospital and ED at a higher rate does not mean it is an important point of care. It does make the hospital an ideal location where one can approach PWUAD at a time when they are feeling vulnerable and provide them options. This makes line 78 even more relevant. | Line 74 and 75 have been amended and the sentence now states that the hospital is an ideal point of care and not an important point of care. (page 3, line 82) |
| **Reviewer #2 Comments** | **Authors’ Response** |
| The inclusion of alcohol interventions is an interesting one. People who use drugs and alcohol as a term is not well known/used, and the inclusion of people who use alcohol certainly can skew/broaden the patient population since the prevalence of alcohol is high. I think the article could do with clarification about the use of this novel term and justifications as to why. Alcohol harm reduction strategies are a relatively newer field and the inclusion of alcohol harm reduction (managed alcohol, BNI, etc) should be explicated. | Thank you for your comment. Based on our review people who use drugs and/or alcohol is not a novel or uncommonly used term. It is common in the field of harm reduction research. Literature using this or similar terms date to at least 2004 and this term has been in use as of 2022. Please refer to this selection of literature for reference:   - <https://doi.org/10.1177/02692163211061994> - <https://doi.org/10.1186/s12954-020-00366-3> - DOI 10.1007/s10461-013-0587-9 - http://www.biomedcentral.com/1471-2458/12/312 - <https://doi.org/10.1089/10872910050046340>   Harm reduction strategies for alcohol have been in use and researched as early as 1983. Literature related to this topic also continues to be published. Please refer to this selection of literature for reference:   - DOI: 10.1080/09595230600944529 - [https://doi.org/10.1016/j.drugpo.2006.05.004](https://doi-org.ezproxy.library.dal.ca/10.1016/j.drugpo.2006.05.004) - [https://doi.org/10.1037/a0026397](https://psycnet.apa.org/doi/10.1037/a0026397) - <https://doi.org/10.3389/fpubh.2022.855416> |
| I am glad that the study interventions were coded alongside the Hawk principles of harm reduction in healthcare. Certainly some here but appreciate the delineation of the coding process. With some of the harm reduction principle codes, I wonder about the results. i.e. Wakeman et al 2017 multidisciplinary team offering motivational counseling, pharmacotherapy, is that not an example of humanism? Does Dong et al 2020 supervised consumption service intervention not epitomize the principle of autonomy? Etc | We appreciate that textual coding involves a certain level of subjective interpretation. Our research team attempted to enhance the rigor of our coding by having two reviewers independently code the included studies based on Hawk’s principles. Discrepancies between codes were brought to the team, who discussed the studies and assessed which code was appropriate based on their knowledge of the included studies and their expertise. However, we acknowledge that this process is not completely objective, and this has been noted as a limitation within the limitation section. (page 25, line 412-414) |
| Clarifications: Page 16 table 2 – what is Nav Star? Page 10, Table 1. Welch et al. What is “Relay program” please explicate for a general audience | The authors have clarified that the Relay Program (Table 1) and NavStar (Table 2) are the names of harm reduction strategies. The content of these strategies is described in the “Intervention Content” column in Table 2 (page 14 and 16). |
| Clarification: Was any of the grey literature included in the final sample? | No grey literature was included in the final sample. (page 6, line 194-195, and Figure 1). |
| Re Table 4: Barriers and Enablers to Implementation: I found this table difficult to read. There were so many barriers, particularly I the “system and organizational factors.” Could this not be further delineated? It might also be useful to identify any barriers and enablers on the same row, i.e. lack of resources, reported enablers “securing funding” if possible. Many of the system and organization factors I think could be further coded and organized. | The table has been reformatted for clarity and readability. (page 20-22, Table 4)  We have added further description in the Data synthesis and presentation section within the Methods to clarify meaning related types of barriers and enablers. (page 6, line 166-173) |
| Discussion could use some re-work and re-organization. Might consider putting partner reflections before the discussion section. Good section on the summary of the key findings in the study, as well as gaps in comparison to the current harm reduction strategies and or previous literature, but would benefit from further explication of research strategies, areas for more rigorous investigation including methods, as well as clinical, policy and research implications. It would be notable to also mention the distinct policy implications of implementation that are not generalizable across the countries included in this scoping review. | To improve clarity and readability the Partner reflections have been tabulated and moved before the discussion section. (page 23, Table 5) |
| I feel the partner/stakeholder reflections section is also incomplete. Were these direct quotes from participants at CBOs and or PWUD? They are listed out almost like direct quotes rather than summarized. Can they be summarized or organized differently? Line 364: “often feel like they must police their own treatment” (?) unclear what they mean by this. Reads very disorganized/unclear, not as cohesive as the rest of the manuscript | As mentioned above, the Partner reflections have been tabulated and moved before the discussion section to improve clarity and readability.   The partner reflections are not direct quotes, but a summary of reflections of our partners. The table has been renamed and several of the statements have been reworded to clarify this. (page 23, Table 5) |
| **Reviewer #3 Comments** | **Authors’ Response** |
| The manuscript draws up a comprehensive database search to find research papers. However, the manuscript rests on many case reports. Moreover, studies from more than one country are included. This is important because one of the identified factors is organizational limitations. Laws, regulations, organizational structure, work culture, funding, etc., tend to differ in different countries. Also, organization limitation is a vague term and has yet to be defined by the authors. Illicit substance-induced disorders like opiates, amphetamines, marijuana, and alcohol abuse disorders share many socio-economic challenges. Still, many factors may vary, which can impact harm reduction strategies for each illicit substance, such as the level of stigma and the general approach of clinicians toward patients with amphetamine will be different from marijuana. Also, laws about illicit substances vary per country. The laws about other illicit substances within a country can vary, e.g., marijuana. | We agree our comprehensive search is a strength of this review. We would agree that organizational limitation is a vague term, and we would like to note that it was not used as a term in this manuscript or any of the included studies. As mentioned above, we amended the Data synthesis and presentation section within the Methods to include a definition of the term system level and organizational factors. (page 6, line 166-173)  We agree with the reviewer that the quality of a study’s findings is dependent on its study design and that barriers and enablers to implementation may vary depending on the country where implementation takes place. However, we would like to emphasize that this review provides a summary of the scope of the available literature related to our topic which was captured by our search strategy and our inclusion criteria. This review is not a meta-analysis, and we are not assessing the generalizability of the results of the included papers or the extent to which study design may limit the generalizability of the findings within each study. To clarify this, the limitation section has been amended with a sentence to that effect. (page 25, line 411-412) |
| **Reviewer #4 Comments** | **Authors’ Response** |
| Excellent work with this review. You have done remarkable work with this article. Though not easy to conduct study like this you have done phenomenal work with this. | Thank you. |
